# Retrospective validation of MetaSystems’ deep-learning-based digital microscopy platform with assistance compared to manual fluorescence microscopy for detection of mycobacteria

**DOI:** 10.1128/jcm.01069-23

**Published:** 2024-02-01

**Authors:** Claudine Desruisseaux, Conor Broderick, Valéry Lavergne, Kim Sy, Duang-Jai Garcia, Gaurav Barot, Kerstin Locher, Charlene Porter, Mélissa Caza, Marthe K. Charles

**Affiliations:** 1 Division of Medical Microbiology and Infection Control, Department of Pathology and Laboratory Medicine, Vancouver General Hospital, Vancouver Coastal Health, Vancouver, British Columbia, Canada; 2 Faculty of Medicine, Department of Pathology and Laboratory Medicine, The University of British Columbia, Vancouver, British Columbia, Canada; University of Manitoba, Winnipeg, Manitoba, Canada

**Keywords:** automated digital microscopy, artificial intelligence, deep learning, deep neural network, acid-fast bacilli, mycobacteria, AFB fluorescence microscopy, clinical validation

## Abstract

This study aimed to validate Metasystems’ automated acid-fast bacilli (AFB) smear microscopy scanning and deep-learning-based image analysis module (Neon Metafer) with assistance on respiratory and pleural samples, compared to conventional manual fluorescence microscopy (MM). Analytical parameters were assessed first, followed by a retrospective validation study. In all, 320 archived auramine-O-stained slides selected non-consecutively [85 originally reported as AFB-smear-positive, 235 AFB-smear-negative slides; with an overall mycobacterial culture positivity rate of 24.1% (77/320)] underwent whole-slide imaging and were analyzed by the Metafer Neon AFB Module (version 4.3.130) using a predetermined probability threshold (PT) for AFB detection of 96%. Digital slides were then examined by a trained reviewer blinded to previous AFB smear and culture results, for the final interpretation of assisted digital microscopy (a-DM). Paired results from both microscopic methods were compared to mycobacterial culture. A scanning failure rate of 10.6% (34/320) was observed, leaving 286 slides for analysis. After discrepant analysis, concordance, positive and negative agreements were 95.5% (95%CI, 92.4%–97.6%), 96.2% (95%CI, 89.2%–99.2%), and 95.2% (95%CI, 91.3%–97.7%), respectively. Using mycobacterial culture as reference standard, a-DM and MM had comparable sensitivities: 90.7% (95%CI, 81.7%–96.2%) versus 92.0% (95%CI, 83.4%–97.0%) (*P*-value = 1.00); while their specificities differed 91.9% (95%CI, 87.4%–95.2%) versus 95.7% (95%CI, 92.1%–98.0%), respectively (*P*-value = 0.03). Using a PT of 96%, MetaSystems’ platform shows acceptable performance. With a national laboratory staff shortage and a local low mycobacterial infection rate, this instrument when combined with culture, can reliably triage-negative AFB-smear respiratory slides and identify positive slides requiring manual confirmation and semi-quantification.

**IMPORTANCE:**

This manuscript presents a full validation of MetaSystems’ automated acid-fast bacilli (AFB) smear microscopy scanning and deep-learning-based image analysis module using a probability threshold of 96% including accuracy, precision studies, and evaluation of limit of AFB detection on respiratory samples when the technology is used with assistance. This study is complementary to the conversation started by Tomasello et al. on the use of image analysis artificial intelligence software in routine mycobacterial diagnostic activities within the context of high-throughput laboratories with low incidence of tuberculosis.

## INTRODUCTION

Early and accurate detection of mycobacterial infections in particular tuberculosis (TB) disease is crucial for clinical management, treatment, and infection prevention and control decisions. Despite major advances in molecular diagnosis, the detection of acid-fast bacilli (AFB) by manual fluorescence microscopy (MM) remains standard practice in both high- and low-prevalence TB settings (1–3). AFB smear microscopy provides information on the morphology (size, width, and length) and arrangement (beading, branching, or cording) of detected acid-fast organisms. When detected, acid-fast bacilli (AFB) are quantified and reported based on a semi-quantitative scoring system (2, 3). This information helps clinicians gauge the level of infectivity and monitor response to treatment (1). While MM is a fast and inexpensive screening method, it is time and labor- intensive, and its accuracy is operator-dependent (1–3).

In an attempt to mitigate the critical workforce shortage, laboratory automation has been considered and implemented in various clinical laboratory areas, such as anatomical pathology, where automated digital microscopy (DM) platforms are starting to be integrated in a routine diagnostic workflow (4, 5). By contrast, in medical microbiology, while many tasks are microscopy based, adoption of such platforms remains limited.

The use of DM for microscopic detection of AFB has been explored through several proof-of-concept studies (6–12). More recently, solutions pairing computer vision artificial intelligence (AI) and DM systems have been made commercially available (13–17). MetaSystems (Altlussheim, Germany), an established manufacturer in DM, has launched a fully automated platform providing microbiology microscopy features including a AFB detection software. Image acquisition of AFB slides and analysis is carried out by the proprietary software (Metafer) which features a deep neural network (DNN) pre-trained by the manufacturer through supervised learning to recognize and segregate objects suspicious of AFB based on a probability score (Fig. S1). MetaSystems assisted digital microscopy (a-DM) platform; in its current state and accordance with its European Union approval, requires final confirmation of AFB-positive results by trained digital reviewers.

The working hypothesis of this study was that conventional MM and a-DM would have equivalent performance and that the latter could be used as a replacement within a routine mycobacterial testing workflow. The primary objectives of this laboratory-based assessment were to determine the analytical performance of the instrument and to assess the AFB smear diagnostic concordance and accuracy of Metafer software a-DM, for respiratory and pleural samples compared to MM. The secondary objective was to establish the reliability of the software’s AFB grading score capacity.

## MATERIALS AND METHODS

### Study setting

This retrospective study was conducted in an academic tertiary care center in Vancouver, British Columbia (Canada), with an annual TB incidence rate of 7.0 per 100,000 population in 2019 (18). It was conducted as a quality improvement project under the University of British Columbia Office of Research Ethics.

### Local standard procedures

#### Sample processing

All lower respiratory or pleural samples submitted for routine mycobacterial testing were decontaminated and/or concentrated on site, excluding samples from previously identified TB cases which were processed in a reference public health laboratory as per local procedure (3, 19). Based on volume, pleural fluids were smeared as neat samples. Each sample was smeared over a surface area of approximately 2.25 cm^2^ on a clean glass slide, stained with auramine-O, and counterstained with 0.5% potassium permanganate (Oxoid, Cambridge, UK, or BD BBL, Sparks, USA). All slides were stored, protected from ambient light, at room temperature (3, 19). Sample processing and staining were identical for slides read by MM and DM.

#### Manual microscopy AFB smear examination

All AFB smears were originally read by one of 13 rotating AFB microscopists using a 40× objective. A minimum of three minutes and 55 fields were required before reporting a smear as negative (3). All new AFB-smear-positive cases were confirmed by a second reviewer [a board-certified Medical Microbiologist (i.e., a clinical microbiologist with medical (M.D.) training)]. Semi-quantification of detected organisms was done by averaging the number of AFB observed per microscopic field (3) (Table S1). Slides with only 1–2 AFB observed for the entire slide were reported as AFB-smear-negative (Table S1).

#### Reference standard

All submitted samples were set up for mycobacterial cultures using a liquid media (Mycobacterial Growth Indicator Tube [MGIT], Beckton-Dickinson, Sparks, USA) and a solid media [Löwenstein-Jensen (LJ), Remel, Lenexa, Kansas, USA]. MGIT tubes were incubated as per the manufacturer’s instructions in an automated mycobacterial detection instrument (BD BACTEC MGIT, Beckton Dickinson, Sparks, USA) for 42 days. LJ was incubated at 37°C ± 1–2°C for a minimum of 8 weeks.

### Digital microscopy parameters

#### Slide scanning and image analysis

Images from auramine-O-stained slides were captured by the high-resolution camera (CoolCube 4th Generation 12 MP camera 12Mega 1.1′ CMOS Color Chip, image size 4,096 × 3,000 pixels; pixel size 3.45 μm × 3.45 µm) using the 20× objective lens. Whole-slide imaging (WSI) protocol was selected, which resulted in a constant number of 420 captured fields (or an equivalent of 586 FoV at 400× magnification) with an image resolution of 0.173 microns per pixel (greater than the minimal digital effective resolution of 0.25 micron per pixel required to view AFB at 40×) (4). A failed scan was defined by the absence of image analysis output for a whole slide after four attempts of scanning. A successful scanning rate of ≥90% was considered acceptable for this study (4).

#### Determination of a probability threshold for AFB detection

For each slide scanned, a total of 234,400 image tiles were individually analyzed by a pre-trained DNN algorithm specific to the Metafer software. Once classified, tiles were displayed on an image gallery and sorted according to a DNN probability threshold (PT) of containing a true AFB. For this validation, the PT was increased from 50% (default setup) to 96% based on a previous pre-commercialization study by Horvath and colleagues and the local sample processing procedure (14).

#### Evaluation of analytical performance

A stock saline solution of *M. tuberculosis* strain H37Rv and *M. avium* strain TMC 724, at 1.0 McFarland standard was prepared (20, 21). From each strain, a dilution series was prepared for the assessment of the limit of detection (LoD) of DM. Slides were prepared by transferring 50 µL from each dilution on WASP slides (COPAN Diagnostics, Murrieta, California, USA) with a predefined smear area. Each slide underwent MM followed by DM on three different days for a total of 15 replicates per dilution tested (30 replicates for 1:128 dilution). LoD was determined as the dilution for which 95% of replicates were detected and LoD_DM_ was expected to be at least equal to LOD_MM_ (22).

#### Repeatability and reproducibility

Repeatability and reproducibility were assessed using the DM scans from MTB dilution 1:64 (corresponding to approximately 3,078 CFU/mL) in saline and negative control (saline) (20, 21). Repeatability was calculated as the percentage of agreement between replicate slides scanned on the same day with the expected result. Reproducibility was shown as the percentage of agreement of all replicate scans of the same slide with the expected result. Repeatability and reproducibility were considered acceptable if ≥95% (22).

### Clinical validation study

#### Clinical sample selection

Between 31 August 2021 and 25 May 2022, 320 clinical samples were considered for inclusion. Sample types included sputa, tracheal aspirates (TA), bronchial washings (BW) or bronchoalveolar lavages (BAL), and pleural fluids (PF). Anonymized slides were selected based on their reported AFB semi-quantitative grading by MM: a minimum of 15 slides per grading and 200 negative slides were included in a non-consecutive manner (3). Slides were excluded if they were found to be damaged.

#### Assisted digital microscopy AFB smear examination

**Fig 1:**
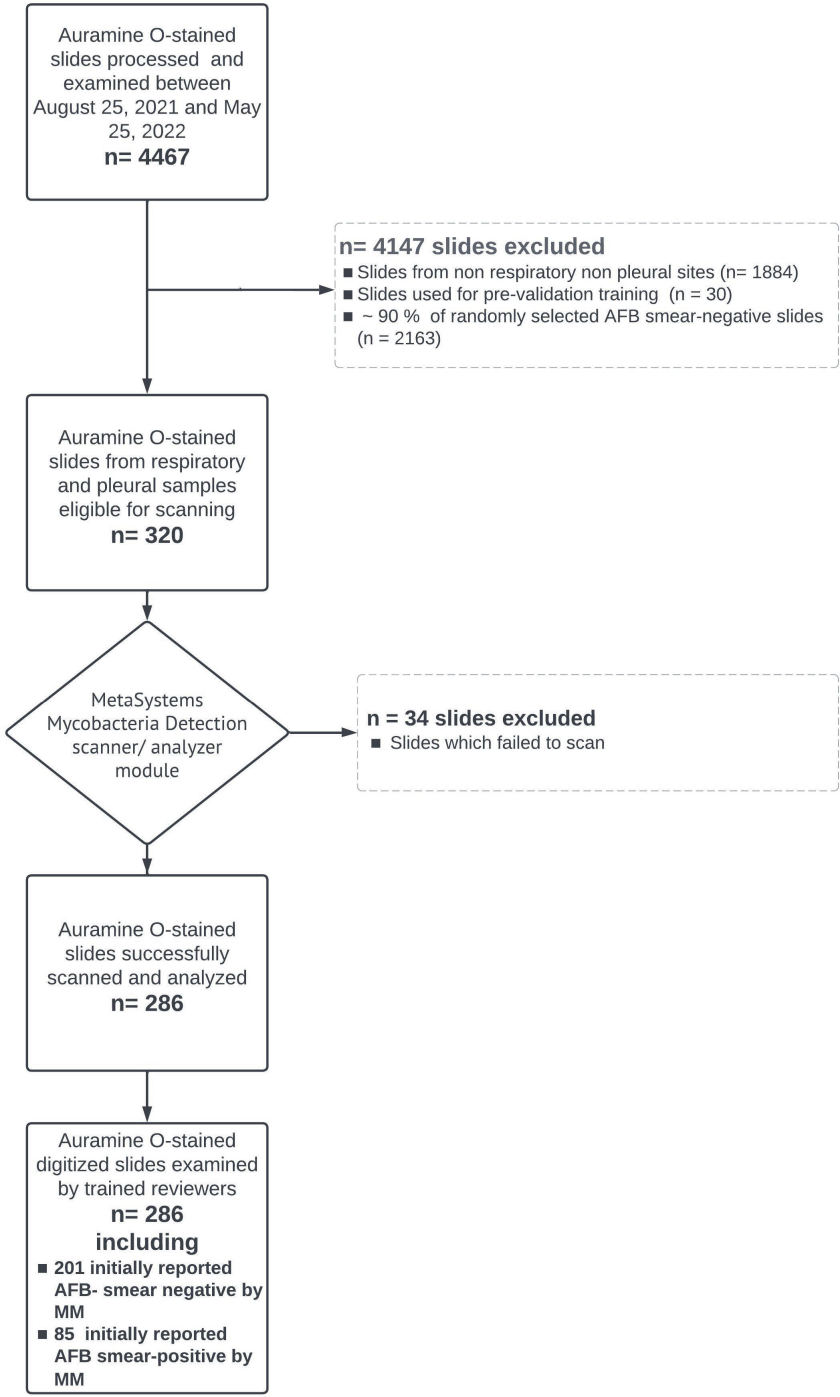
Flowchart diagram summary of the clinical validation study.

Three digital reviewers (including a senior technologist with more than 1,000 hours of experience in AFB smear microscopy) received a 1-hour training session, and a minimum of 30 slides were used for pre-validation training (Fig. 1). Trained digital reviewers reviewed and interpreted the slide scan in NEON Metafer, providing “assisted-DM” (a-DM) AFB smear status and grading score results. All reviewers were blinded to the original MM reporting, other reviewers’ a-DM, and culture results. Slide reviews were performed independently. In the event of disagreements between reviewers, as per the local protocol, results from the medical microbiologist’s review served as a reference and were thus considered for definitive analysis (4).

In the software, digital slides with tiles containing only AFB objects detected at a PT <96% were resulted by reviewers as AFB smear negative without further action. Slides with only one or two positive AFB objects at a PT ≥96% confirmed by reviewers initially resulted in a doubtful AFB smear status (Table S1). Digital slides with more than three confirmed positive objects were considered AFB smear positive. For each AFB-smear-positive slide, a CDC’s semi-quantitative grading score was generated (Table S1). Digital slides with a doubtful AFB smear status result were classified as AFB smear negative for this study.

#### Investigation of discrepant results between a-DM and MM

Concordance was defined by both microscopic methods agreeing upon the presence or absence of AFB. For suspected reading errors, the original slides were re-examined by MM, then re-scanned and reviewed by a-DM. To investigate fluorescence fading, slides initially reported as AFB smear positive by MM and resulted as AFB smear negative by DM were re-stained by cold Ziehl-Neelsen (ZN) technique (Carbolfuchsin, 3% acid-alcohol-Methylene Blue, Remel) (19, 23). Slides with possible or confirmed fading (revealed by ZN) were considered as non-discrepant for the analysis (Table S2).

### Diagnostic concordance and accuracy

#### AFB smear diagnostic concordance

Diagnostic concordance of AFB smear between a-DM and MM was assessed prior to and after discrepant analysis. Overall concordance rate (OCR), positive-percent agreement (PPA), and negative-percent agreement (NPA) between the two methods were calculated. OCR, PPA, and NPA were considered acceptable if ≥90% (22).

#### Comparative diagnostic accuracy: three-way comparison between a-DM, MM, and mycobacterial culture, categorization of diagnostic discordance and differences in accuracy

**Fig 2:**
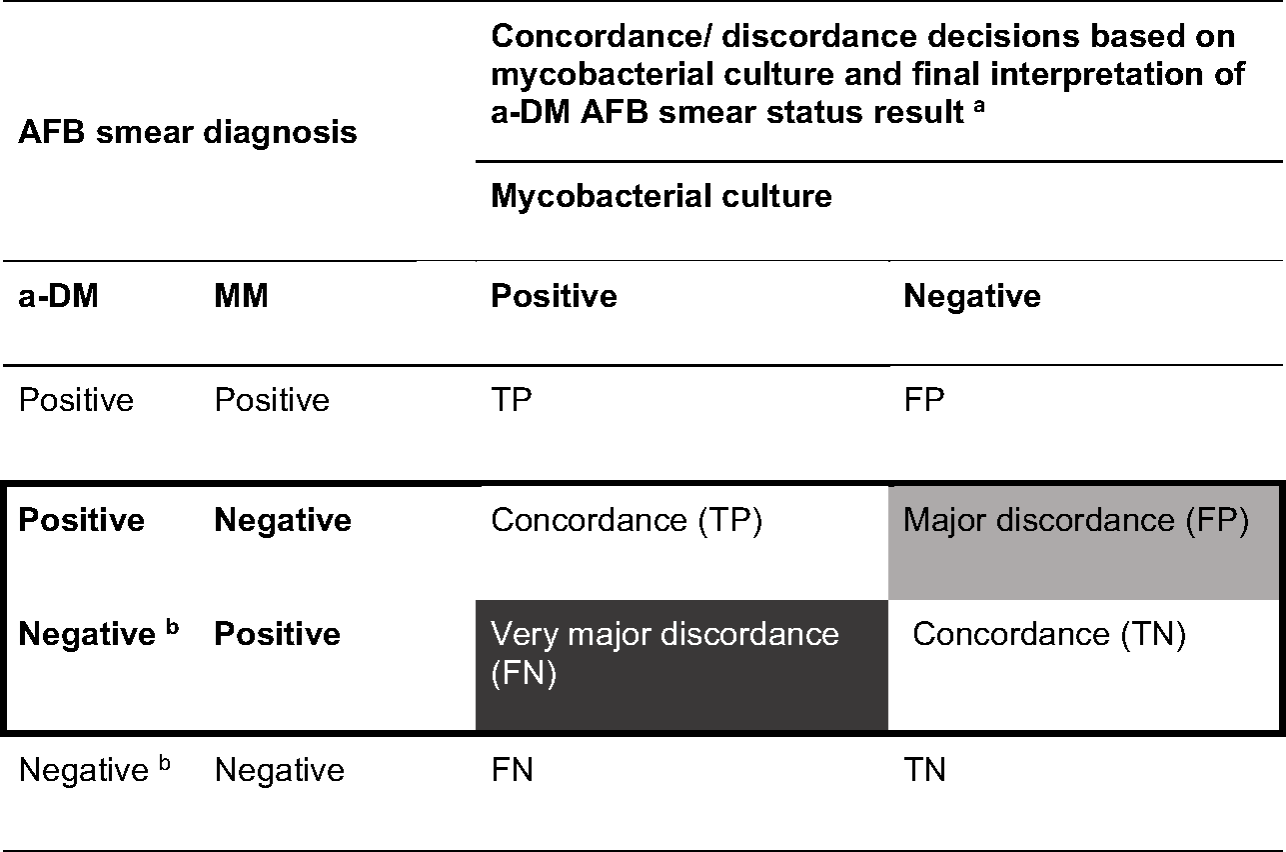
Concordances and discordances decisions for discordant paired AFB smear diagnoses based on mycobacterial culture as the diagnostic accuracy criteria. ^a^ TP: true positive; FP: false positive; TN: true negative; FN: false negative; ^b^ Slides with only 1–2 confirmed AFB objects were initially considered doubtful and ultimately interpreted as smear negative.

Using mycobacterial culture as the reference standard, sensitivity and specificity were assessed for each microscopic modality. To compare differences in accuracy, a post-discrepant analysis three-way comparison of results was conducted as per the CLSI EP12 guidelines; each paired AFB smear status results (MM and a-DM) were compared and interpreted in function of the culture result (Fig. 2) (22). Sensitivity and specificity were estimated for each microscopic modality and were considered acceptable if ≥90%. Differences in paired sensitivities (i.e., sensitivity_a-DM_ – sensitivity_MM_) and specificities (i.e., specificity_a-DM_ – specificity_MM_) were measured (22).

#### AFB grading score agreement

Grading score agreement between a-DM and MM was achieved when both methods agreed according to CDC’s semi-quantitative AFB grading score. For each semi-quantitative grading score, agreement estimates were calculated. Agreement was considered acceptable if ≥0.90 (22).

### Statistical analysis

Statistical analyses were performed using GraphPad Prism 9th Edition (GraphPad Software, San Diego, CA, USA). 95% confidence intervals (CI) were calculated using the Wilson score method. McNemar test was used to assess differences in paired sensitivities and specificities with an α-value of 0.05 (24, 25).

## RESULTS

### Digital microscopy parameters

#### Analytical performance of DM

AFB were reliably detected down to 1:64 dilution (approximately 3,078 CFU/ mL) in 96% (43/45 replicates) and 100% (30/30 replicates) by DM and MM, respectively. Using known dilutions of MTB, both MM and DM were found to be equally sensitive and demonstrated consistent AFB detection down to a dilution of 1:64. Using known dilutions of MAC, the limit of detection of DM was estimated to be inferior (between 1:16 and 1:32 dilutions) to that of MM (estimated at 1:64 dilution) (Table S3a).

#### Repeatability and reproducibility

The (intra-run) repeatability and (inter-run) reproducibility of DM were found to be 100% concordant when 1:64 dilution of MTB and negative control were tested (Table S3b).

### Clinical validation

#### Characteristics of samples for the clinical validation

A total of 286 slides were included for clinical validation (Fig. 1) with bronchoscopy samples representing 50.6% of scanned slides (Table 1). As per MM, 201 slides were originally reported as AFB smear negative and 85 were AFB smear positive. Of the latter, 72 (84.7%) had a corresponding positive culture for *Mycobacterium tuberculosis* complex (MTBC, *n* = 37), *Mycobacterium avium* complex (MAC, *n* = 22), or other nontuberculous mycobacteria (NTM) species (*n* = 13). Of the 201 AFB smear-negative slides by MM, three had a corresponding positive culture (MAC, *n* = 2; MTBC, *n* = 1).

**TABLE 1:** Characteristics of clinical validation slides^*a*^

Characteristics	n
Scanned slides	320
Excluded slides due to scanning failure, n (%)	34 (10.6%)
Previously reported AFB-smear-negative slides	34
Sputum or tracheal aspirates, n/ no. of scanned sputum/TA (%)	10/114 (8.7 %)
BAL or BW, n/ no. of scanned BAL or BW (%)	16/161 (9.9 %)
Pleural fluid, n/ no. of scanned pleural fluid (%)	8/45 (17.8%)
Previously reported AFB-smear-positive slides	0
Included slides	286
Previously reported AFB-smear-negative slides	201
Previously reported AFB-smear-positive slides	85
1+	28
2+	21
3+	14
4+	22
Age of slides at time of scanning [average ±standard deviation (days)]	63.3 ± 75.2
Average number of scanning events for acceptance	1.1 ± 0.3
Average time of scanning and image analysis per whole slide (minutes)	14.0
Sample types	
Sputum or tracheal aspirate, n (%)	104 (36.3%)
BAL or BW, n (%)	145 (50.6%)
Pleural fluid, n (%)	37 (12.9%)
Slides with corresponding culture-negative samples	211/286 (73,4%)
Slides with corresponding culture-positive samples	75/286 (26.2 %)
MTBC	38/75 (50.7%)
MAC	24/75 (32.0%)
Other NTM	13/75 (17.3%)

^*a*^ no., number; AFB, acid-fast bacilli; BAL, broncho-alveolo-lavage; BW: bronchial washings; MTBC, *Mycobacterium tuberculosis* complex; MAC, *Mycobacterium avium* complex; NTM, non-tuberculous mycobacteria.

#### Assisted-digital microscopy AFB smear examination

At the time of initial DM scanning, the mean age of slides was 63 days [Standard deviation (SD ) = 75]. An overall scan failure rate of 10.6% (34/320) was recorded. Failures to scan were caused by the scanner’s inability to find a focus plane and were exclusively observed in clinical slides originally reported as AFB smear negative by MM; primarily from pleural and bronchoscopy samples (Table 1). Out of the 286 successfully scanned slides, the majority of slides (96.5%, 276/286) were adequate for analysis after the first run of scanning.

Of the 204 slides reported as AFB smear negative by DM, 14 slides were initially reviewed as having a doubtful AFB smear status. Of the 82 AFB-smear-positive slides, the final AFB semi-quantitative grading was distributed as follows: 3.7% (3/82) graded at 4+, 18.3% (15/82) at “3+,” 58.5% (48/82) at “2+,” and 19.5% of slides (16/82) at “1+” (Table S4).

When reproducibility was assessed using a subset of five AFB-smear-positive and five AFB-smear-negative clinical validation slides, a-DM demonstrated agreement of 93% and 100%, respectively (Table S3b).

#### Investigation of discrepant AFB smear status results between MM and a-DM

**Fig 3:**
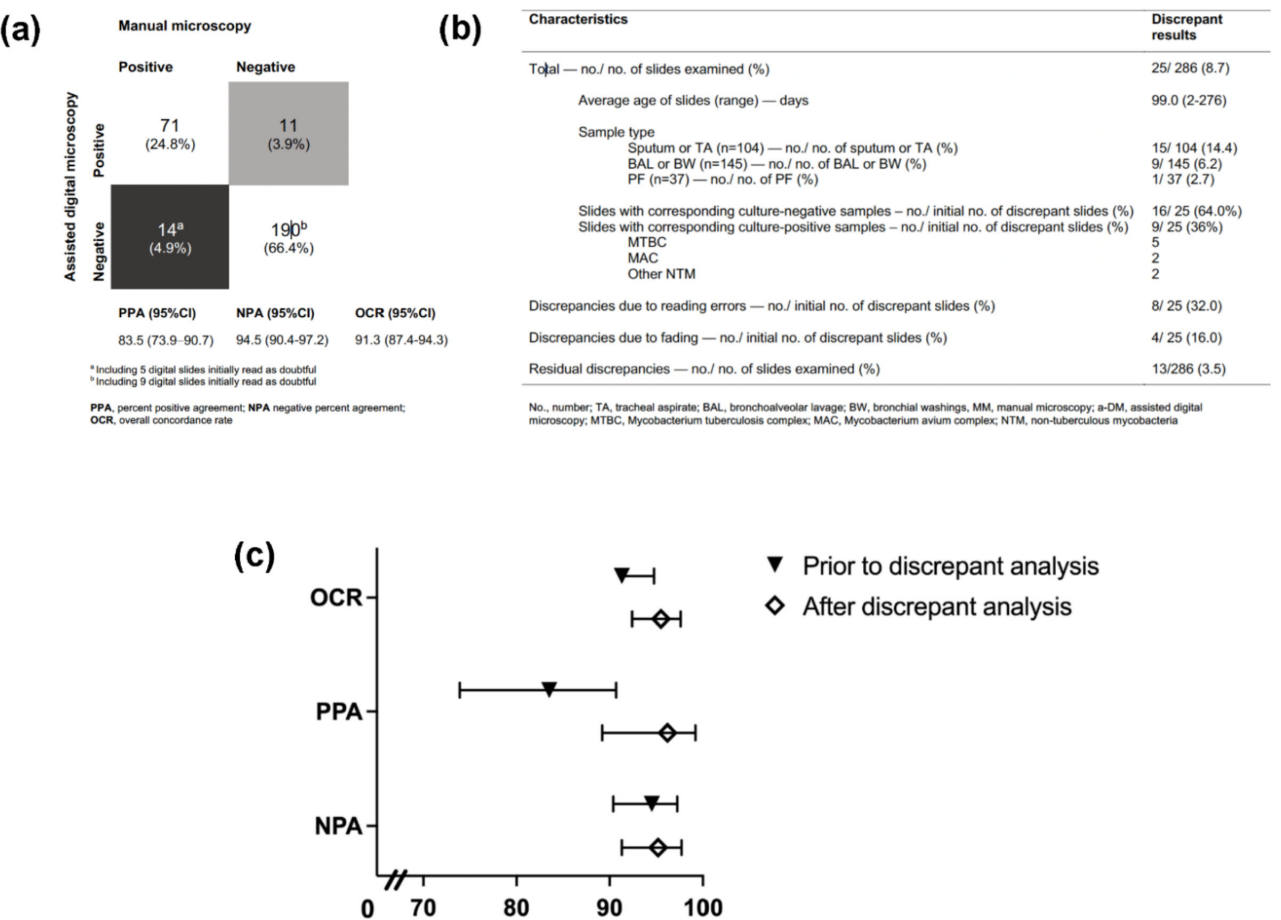
(a) Diagnostic AFB smear concordance between a-DM and MM prior to discrepancy analysis. (b) Investigation of discrepant results between both methods. (c) Comparative overall concordance rate, positive-percent agreement (PPA), and negative-percent agreement (NPA) between both methods prior to and following discrepant analysis. PPA, percent positive agreement; NPA, negative percent agreement; OCR, overall concordance rate; No., number; TA, tracheal aspirate; BAL, bronchoalveolar lavage; BW, bronchial washings; MM, manual microscopy; a-DM, assisted digital microscopy; MTBC, *Mycobacterium tuberculosis* complex; MAC, *Mycobacterium avium* complex; NTM, non-tuberculous mycobacteria.

A total of 25 (8.7%) slides showed discrepant results between a-DM and MM (Fig. 3). The majority of discrepant results (15/25) occurred in slides from sputum and tracheal aspirate samples with an average age of 93 days. Eight (32%) discrepancies were resolved following repeat AFB examination by both MM and a-DM (Fig. 3a) and four discrepant results were likely caused by the fading of auramine-O (including one confirmed by ZN re-staining) (Table S5). Following investigation, 13 (3.5%) results remained discrepant (a-DM smear positive/MM smear negative, *n* = 10; a-DM smear negative/MM smear positive, *n* = 3) (Table S5).

### Diagnostic concordance and accuracy

#### Diagnostic AFB smear concordance (a-DM vs MM)

The initial analysis of a-DM to MM showed comparable results (Fig. 3a). Following discrepant analysis, recalculated PPA and NPA of a-DM increased to 96.2%, 95% CI (89.2%–99.2%) and 95.2% (95%CI, 91.3%–97.7%), respectively, for an overall concordance of 95.5% (95%CI, 92.4%–97.6%) (Fig. 3c).

#### Comparative diagnostic accuracy

**Fig 4:**
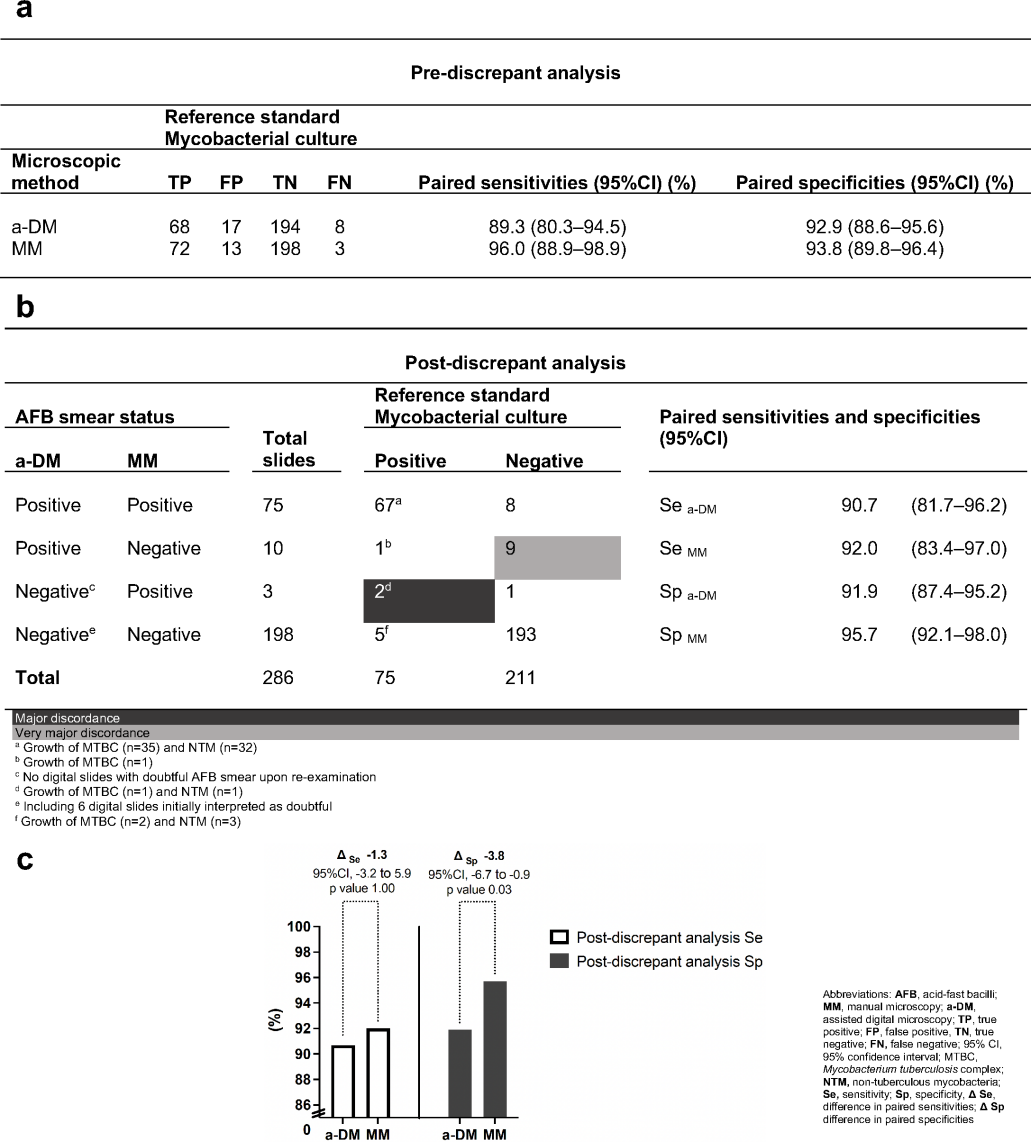
(a) Comparative paired diagnostic accuracy of a-DM and MM compared to mycobacterial culture before discrepant analysis. (b) Three-way comparison table between a-DM, MM, and mycobacterial culture following discrepant analysis. (c) Post-discrepant analysis differences in paired sensitivities and specificities of both microscopic methods with 95% Cl and *P* value according to McNemar’s test. ^a^ Growth of MTBC (*n* = 35) and NTM (*n* = 32); ^b^ Growth of MTBC (*n* = 1); ^c^ All digital slides initially observed to be AFB negative upon re-examination; ^d^ Growth of MTBC (*n* = 1) and NTM (*n* = 1); ^e^ Including six digital slides initially interpreted as doubtful; ^f^ Growth of MTBC (*n* = 2) and NTM (*n* = 3). Abbreviations: AFB, acid-fast bacilli; MM, manual microscopy; a-DM, assisted digital microscopy; TP, true positive; FP, false positive; TN, true negative, FN, false negative; 95% CI, 95% CI; MTBC, *Mycobacterium tuberculosis* complex; MAC, *Mycobacterium avium* complex; NTM, non-tuberculous mycobacteria; Se, sensitivity; Sp, specificity; Δ Se, difference in paired sensitivities; ΔSp: difference in paired specificities.

Following discrepant analysis, 273 (95.5%) of 286 pairs of AFB smear diagnoses were concordant (Fig. 4a). Of these, 260 were in keeping with the mycobacterial culture results (i.e., 67 true positives and 193 true negatives). Among the 13 discordant AFB diagnostic pairs, very major discordances were observed in two slides with corresponding mycobacterial growth (MTBC *n* = 1, NTM *n* = 1). Major discordances were mostly graded AFB smear 1 + by a-DM (5/9) (Table S5). For only 2 of 13 discordant pairs, a-DM outperformed MM as per mycobacterial culture results. Representative images of AFB-object detected by the software in concordant and discordant AFB diagnostic pairs are exemplified in Fig. S2.

After discrepant analysis, a sensitivity of a-DM increased to 90.7% (95%CI, 81.7%–96.2%), whereas the specificity decreased to 91.9% (95%CI, 87.4%–95.2%) (Fig. 4b). The difference between paired sensitivities of a-DM when compared to MM was −1.3% (95%CI, −3.2 to 5.9%), *P*-value = 1.00). A statistically significant difference in paired specificities was estimated at −3.8% (95%CI, −6.7 to −0.9%), *P*-value = 0.03 (Fig. 4c).

#### AFB grading score agreement

Overall, the AFB grading score agreement between a-DM and MM was 74.8% (214/286) (Table S4). Compared to MM, a-DM classified correctly only 3/22 (13.6%) AFB-smear-positive slides originally reported as 4, and 5/14 (35.7%) originally reported as 3+. Of the 21 slides previously graded as 2 + by MM, 17 (81.0 %) were appropriately classified by a-DM.

## DISCUSSION

### Diagnostic concordance and accuracy

This retrospective validation study assessed the binary output performance of MetaSystems’ platform on respiratory and pleural samples, compared to conventional MM. The results met the predefined criteria of acceptability. The concordance, specificity, and sensitivity of a-DM were set at ≥90% differing from the recommended concordance threshold (≥95%) by CAP for digital pathology (5). The acceptability threshold was lowered since fluorescence microscopy is primarily used as a screening method in the mycobacterial infection diagnostic and management algorithm; ultimately, culture and identification are required for therapy initiation.

Comparable concordance rates of 92.7% (16) and 95.7% (26) with MM have been described in recent studies evaluating other AI-powered automated AFB microscopy systems in high TB incidence settings. Interestingly, Tomasello et al. observed a drop in sensitivity (from 97.0% to 70.7%) when a similar version of the MetaSystems’ AFB detection software was employed with assistance at a DNN PT of 50% (27). According to the authors, images captured out-of-focus made it difficult to distinguish AFB from artifacts, causing operators to falsely interpret slides as AFB smear negative (27). Differences in a-DM’s sensitivity between this study and Tomasello’s may be related to the sample types used for analysis. A greater proportion of slides from non-respiratory samples were analyzed in Tomasello’s study (43.7%) compared to the present study (12.9%); non-respiratory samples exhibit variability in cellularity and background debris, shown to impact automated focus capabilities and ultimately digital review (4, 27). Furthermore, when operating MetaSystem’s DDN software mainly pre-trained to recognize AFB from MTBC (14), it was observed that the definition of AFB objects from NTM-positive samples differed from MTBC-positive samples, thus influencing review by the operator. As such, this may explain the higher sensitivity (90.7%) estimated in this study versus Tomasello and colleagues, where MTBC was recovered in 50.7% (38/75) versus 24.1%(101/133) of slides with corresponding mycobacterial growth, respectively. Nonetheless, Tomasello et al. have reported an overall a-DM specificity comparable to the hereby reported a-DM specificity (27). A very limited number of studies evaluating MetaSystems’ platform as a standalone image analysis AI instrument showed that the performance of a given DNN classifier algorithm varies according to pre-set cut-off for object classification, with optimal trades off between sensitivity/specificity at a PT ≥95% (14, 27, 28). The findings of this validation study are in keeping with the previously reported conclusions on the impact of the PT value. Furthermore, it is probable that adjusting the PT helps achieve optimal performance in the function of the sample type (27).

### AFB grading scoring capacity

When the AFB grading agreement was investigated, a poor agreement of <40% was found for originally highly positive AFB smears (i.e., 3 + and 4+). To appropriately compare AFB grading scores, the MetaSystems DNN classifier was tailored to determine a score according to the average number of positive AFB objects per tile with PT ≥96% per field of view. Thus, the WSI protocol used also impacted the overall AFB grading result. In addition, the software considered each positive tile as one distinct positive AFB object irrespective of the number of bacilli visible within a tile. Finally, fluorescence fading may constitute another contributing factor influencing poor AFB grading agreement in high-burden AFB smears.

### Strengths and limitations

This study was a full validation of Metafer software comprising AFB smear diagnostic concordance, accuracy, precision studies, and limit of AFB detection assessments, and complied with recommendations from the Standards for Reporting Diagnostic Accuracy Studies (STARD) (29, 30).

It is important to note that this laboratory-based study did not include clinical data and mycobacterial culture was used as the sole reference standard to compare results yielded by each microscopic modality, likely influencing estimates of sensitivity and specificity in this study. The non-consecutive slide selection done to maximize the number of AFB-smear-positive samples and the inclusion of an undetermined proportion of slides from patients on antimycobacterial therapy constitute additional factors impacting sensitivity and specificity respectively.

The ratio of slides from initial diagnostic versus follow-up samples in the present study was influenced by local standard procedures (involving slides from known TB cases not processed in-house) and likely differs from those observed elsewhere. Other factors possibly affecting the generalizability of both results and DM setup parameters to other laboratory settings involve the over-representation of slides from bronchoscopy samples (although in keeping with the local proportion of samples) and the high proportion (87%, 249/286) of slides which stemmed from processed samples.

Other limitations include the limited number of pleural fluids (< 60) evaluated (below the recommended number for validation) (5). In addition, a relatively high scanning failure rate is attributable to the WSI scanner’s inability to find focus planes in slides with limited cellularity or with little positive auramine-O staining material. While high WSI scan failure rates have been described in a similar subset of slides, this may be mitigated by standardization of the smear area (i.e., use of glass slides with a pre-defined smear area) (31). While mimicking real-laboratory conditions, another limitation was that MM AFB smear status and grading score results were generated by several technologists, causing interrater variability. Finally, the retrospective nature of this study using archived auramine-O slides subject to fading is another limiting consideration. The use of older slides may explain a certain proportion of initial discrepancies observed.

### Conclusion

This comparative accuracy study supports the use of MetaSystems’ platforms as a triage method complementary to conventional manual microscopy in respiratory samples. When using a 96% DNN probability threshold, an AFB-smear-negative slide could be rapidly identified by the instrument with high-level confidence and minimal intervention by a trained digital reviewer. Meanwhile, with its current performance, detected AFB-smear-positive slides would require review by manual microscopy for confirmation and semi-quantification. The adoption of such technology within an established AFB testing algorithm could help streamline the use of molecular detection assays, where nucleic acid amplification tests would be reserved for AFB-smear-positive samples identified digitally (9, 16, 17). Overall, a-DM has the potential to improve laboratory productivity by allowing redistribution of the workforce particularly in high-throughput laboratories with low incidence TB. This is consistent with the envisaged role of AI-based DM platforms in other smear-based microbiological diagnostic fields (28, 32–35). Further enhancement of the current version of the DNN algorithm of the software is required to achieve an acceptable performance with respect to AFB grading score and these may be rapidly achieved through inherent learning capacities of the system and improvement of image analysis software to pixel segmentation (32). Future prospective clinical studies will be required to establish its clinical impact and further assess implementation considerations within a total laboratory automation setting.

## SUPPLEMENTAL MATERIAL

Supplemental material jcm.01069-23-s0001.pdf Tables and figures supporting data.

ASM does not own the copyrights to Supplemental Material that may be linked to, or accessed through, an article. The authors have granted ASM a non-exclusive, world-wide license to publish the Supplemental Material files. Please contact the corresponding author directly for reuse.

## References

[1] Behr MA, Lapierre SG, Kunimoto DY, Lee RS, Long R, Sekirov I, Soualhine H, Turenne CY. 2022. Chapter 3: diagnosis of tuberculosis disease and drug-resistant tuberculosis. Can J Respir Crit Care Sleep Med 6:33–48. doi:10.1080/24745332.2022.2035638

[2] Forbes BA, Hall GS, Miller MB, Novak SM, Rowlinson M-C, Salfinger M, Somoskövi A, Warshauer DM, Wilson ML. 2018. Practical guidance for clinical microbiology laboratories: mycobacteria. Clin Microbiol Rev 31:e00038-17. doi:10.1128/CMR.00038-17 29386234 PMC5967691

[3] CLSI. 2018. CLSI document M48-A2. Laboratory detection and identification of mycobacteria; approved guideline. Clinical and Laboratory Standards Institute, Wayne, PA.

[4] García-Rojo M. 2016. International clinical guidelines for the adoption of digital pathology: a review of technical aspects. Pathobiology 83:99–109. doi:10.1159/000441192 27100834

[5] Evans AJ, Brown RW, Bui MM, Chlipala EA, Lacchetti C, Milner DA, Pantanowitz L, Parwani AV, Reid K, Riben MW, Reuter VE, Stephens L, Stewart RL, Thomas NE. 2022. Validating whole slide imaging systems for diagnostic purposes in pathology. Arch Pathol Lab Med 146:440–450. doi:10.5858/arpa.2020-0723-CP 34003251

[6] Somoskövi A, Györi Z, Czoboly N, Magyar P. 1999. Application of a computer-directed automated microscope in mycobacteriology. Int J Tuberc Lung Dis 3:354–357. 10206508

[7] Lewis JJ, Chihota VN, van der Meulen M, Fourie PB, Fielding KL, Grant AD, Dorman SE, Churchyard GJ. 2012. "Proof-of-concept" evaluation of an automated sputum smear microscopy system for tuberculosis diagnosis. PLoS One 7:e50173. doi:10.1371/journal.pone.0050173 23209666 PMC3510232

[8] Panicker RO, Soman B, Saini G, Rajan J. 2016. A review of automatic methods based on image processing techniques for tuberculosis detection from microscopic sputum smear images. J Med Syst 40:17. doi:10.1007/s10916-015-0388-y 26573654

[9] Nabeta P, Havumaki J, Ha DTM, Caceres T, Hang PT, Collantes J, Thi Ngoc Lan N, Gotuzzo E, Denkinger CM. 2017. Feasibility of the TBDx automated digital microscopy system for the diagnosis of pulmonary tuberculosis. PLoS One 12:e0173092. doi:10.1371/journal.pone.0173092 28253302 PMC5333855

[10] Law YN, Jian H, Lo NWS, Ip M, Chan MMY, Kam KM, Wu X. 2018. Low cost automated whole smear microscopy screening system for detection of acid fast bacilli. PLoS One 13:e0190988. doi:10.1371/journal.pone.0190988 29357378 PMC5777646

[11] Zingue D, Weber P, Soltani F, Raoult D, Drancourt M. 2018. Automatic microscopic detection of mycobacteria in sputum: a proof-of-concept. Sci Rep 8:11308. doi:10.1038/s41598-018-29660-8 30054578 PMC6063956

[12] Chesov D, Lesanu V, Ciobanu N, Codreanu A, Crudu V, Cuevas LE, Kiss D, Czoboly N, Somoskovi A. 2020. Automated high-throughput digital fluorescence microscopy for TB diagnosis. Int J Tuberc Lung Dis 24:1103–1105. doi:10.5588/ijtld.19.0811 33126946

[13] Khare V, Agrawal A, Gupta P, Saxena S. 2019. Artificial intelligence based AFB microscopy for pulmonary tuberculosis in North India: a pilot study. Int J Sci Res Pub 9:9669. doi:10.29322/IJSRP.9.12.2019.p9669

[14] Horvath L, Hänselmann S, Mannsperger H, Degenhardt S, Last K, Zimmermann S, Burckhardt I. 2020. Machine-assisted interpretation of auramine stains substantially increases through-put and sensitivity of microscopic tuberculosis diagnosis. Tuberculosis (Edinb) 125:101993. doi:10.1016/j.tube.2020.101993 33010589

[15] Pantanowitz L, Wu U, Seigh L, LoPresti E, Yeh FC, Salgia P, Michelow P, Hazelhurst S, Chen WY, Hartman D, Yeh CY. 2021. Artificial intelligence-based screening for mycobacteria in whole-slide images of tissue samples. Am J Clin Pathol 156:117–128. doi:10.1093/ajcp/aqaa215 33527136

[16] Fu HT, Tu HZ, Lee HS, Lin YE, Lin CW. 2022. Evaluation of an AI-based TB AFB smear screening system for laboratory diagnosis on routine practice. Sensors (Basel) 22:8497. doi:10.3390/s22218497 36366194 PMC9657727

[17] Huang HC, Kuo KL, Lo MH, Chou HY, Lin YE. 2022. Novel TB smear microscopy automation system in detecting acid-fast bacilli for tuberculosis - A multi-center double blind study. Tuberculosis (Edinb) 135:102212. doi:10.1016/j.tube.2022.102212 35609488 PMC9116043

[18] British Columbia Centre for Disease Control (BC CDC). 2019. Reportable diseases data dashboard: tuberculosis. Available from: http://www.bccdc.ca/health-professionals/data-reports/reportable-diseases-data-dashboard?Disease=Tuberculosis. Retrieved 01 May 2023.

[19] Weitzman I. 2016. Acid-fast stain, p 1–7. In Leber AL (ed), Clinical microbiology procedures handbook, 4th ed. ASM Press, Washington, DC.

[20] Peñuelas-Urquides K, Villarreal-Treviño L, Silva-Ramírez B, Rivadeneyra-Espinoza L, Said-Fernández S, de León MB. 2013. Measuring of Mycobacterium tuberculosis growth. A correlation of the optical measurements with colony forming units. Braz J Microbiol 44:287–289. doi:10.1590/S1517-83822013000100042 24159318 PMC3804212

[21] CLSI. 2018. CLSI standard M24. Susceptibility testing of mycobacteria, Nocardia spp., and other aerobic actinomycetes. 3rd ed. Clinical and Laboratory Standards Institute, Wayne, PA.

[22] CLSI. 2023. CLSI guideline EP12. User protocol for evaluation of qualitative test performance. 3rd ed. Clinical and Laboratory Standards Institue.

[23] Minion J, Shenai S, Vadwai V, Tipnis T, Greenaway C, Menzies D, Ramsay A, Rodrigues C, Pai M. 2011. Fading of auramine-stained mycobacterial smears and implications for external quality assurance. J Clin Microbiol 49:2024–2026. doi:10.1128/JCM.00507-11 21430105 PMC3122634

[24] Trajman A, Luiz RR. 2008. McNemar Chi2 test revisited: comparing sensitivity and specificity of diagnostic examinations. Scand J Clin Lab Invest 68:77–80. doi:10.1080/00365510701666031 18224558

[25] Hayen A, Macaskill P, Irwig L, Bossuyt P. 2010. Appropriate statistical methods are required to assess diagnostic tests for replacement, add-on, and triage. J Clin Epidemiol 63:883–891. doi:10.1016/j.jclinepi.2009.08.024 20079607

[26] Zhang Y, Jiang H, Ye T, Juhas M. 2021. Deep learning for imaging and detection of microorganisms. Trends Microbiol 29:569–572. doi:10.1016/j.tim.2021.01.006 33531192

[27] Tomasello G, Foroughi F, Padron D, Moreno A, Banaei N. 2022. Evaluation of MetaSystems automated fluorescent microscopy system for the machine-assisted detection of acid-fast bacilli in clinical samples. J Clin Microbiol 60:e0113122. doi:10.1128/jcm.01131-22 36121216 PMC9580351

[28] Smith KP, Kang AD, Kirby JE. 2018. Automated interpretation of blood culture gram stains by use of a deep convolutional neural network. J Clin Microbiol 56:e01521-17. doi:10.1128/JCM.01521-17 29187563 PMC5824030

[29] Bossuyt PM, Reitsma JB, Bruns DE, Gatsonis CA, Glasziou PP, Irwig L, Lijmer JG, Moher D, Rennie D, de Vet HCW, Kressel HY, Rifai N, Golub RM, Altman DG, Hooft L, Korevaar DA, Cohen JF, STARD Group. 2015. STARD 2015: an updated list of essential items for reporting diagnostic accuracy studies. Clin Chem 61:1446–1452. doi:10.1373/clinchem.2015.246280 26510957

[30] Cohen JF, Korevaar DA, Altman DG, Bruns DE, Gatsonis CA, Hooft L, Irwig L, Levine D, Reitsma JB, de Vet HCW, Bossuyt PMM. 2016. STARD 2015 guidelines for reporting diagnostic accuracy studies: explanation and elaboration. BMJ Open 6:e012799. doi:10.1136/bmjopen-2016-012799 PMC5128957 28137831

[31] Thrall MJ, Wimmer JL, Schwartz MR. 2015. Validation of multiple whole slide imaging scanners based on the guideline from the college of American pathologists pathology and laboratory quality center. Arch Pathol Lab Med 139:656–664. doi:10.5858/arpa.2014-0073-OA 25927149

[32] Smith KP, Kirby JE. 2020. Image analysis and artificial intelligence in infectious disease diagnostics. Clin Microbiol Infect 26:1318–1323. doi:10.1016/j.cmi.2020.03.012 32213317 PMC7508855

[33] Rhoads DD. 2020. Computer vision and artificial intelligence are emerging diagnostic tools for the clinical microbiologist. J Clin Microbiol 58:e00511-20. doi:10.1128/JCM.00511-20 32295889 PMC7269399

[34] Mathison BA, Kohan JL, Walker JF, Smith RB, Ardon O, Couturier MR. 2020. Detection of intestinal protozoa in trichrome-stained stool specimens by use of a deep convolutional neural network. J Clin Microbiol 58:e02053-19. doi:10.1128/JCM.02053-19 32295888 PMC7269375

[35] Burns BL, Rhoads DD, Misra A. 2023. The use of machine learning for image analysis artificial intelligence in clinical microbiology. J Clin Microbiol 61:e0233621. doi:10.1128/jcm.02336-21 37395657 PMC10575257

