# Supplementary material for: Retrospective validation of MetaSystems’ deep-learning-based digital microscopy platform with assistance compared to manual fluorescence microscopy for detection of mycobacteria
Source: J Clin Microbiol. 2024 Feb 1;62(3):e01069-23. doi: 10.1128/jcm.01069-23 (PMC10935628; doi:10.1128/jcm.01069-23)
Supplement: Supplemental material — Tables and figures supporting data. [file jcm.01069-23-s0001.pdf]

SUPPLEMENTARY MATERIAL

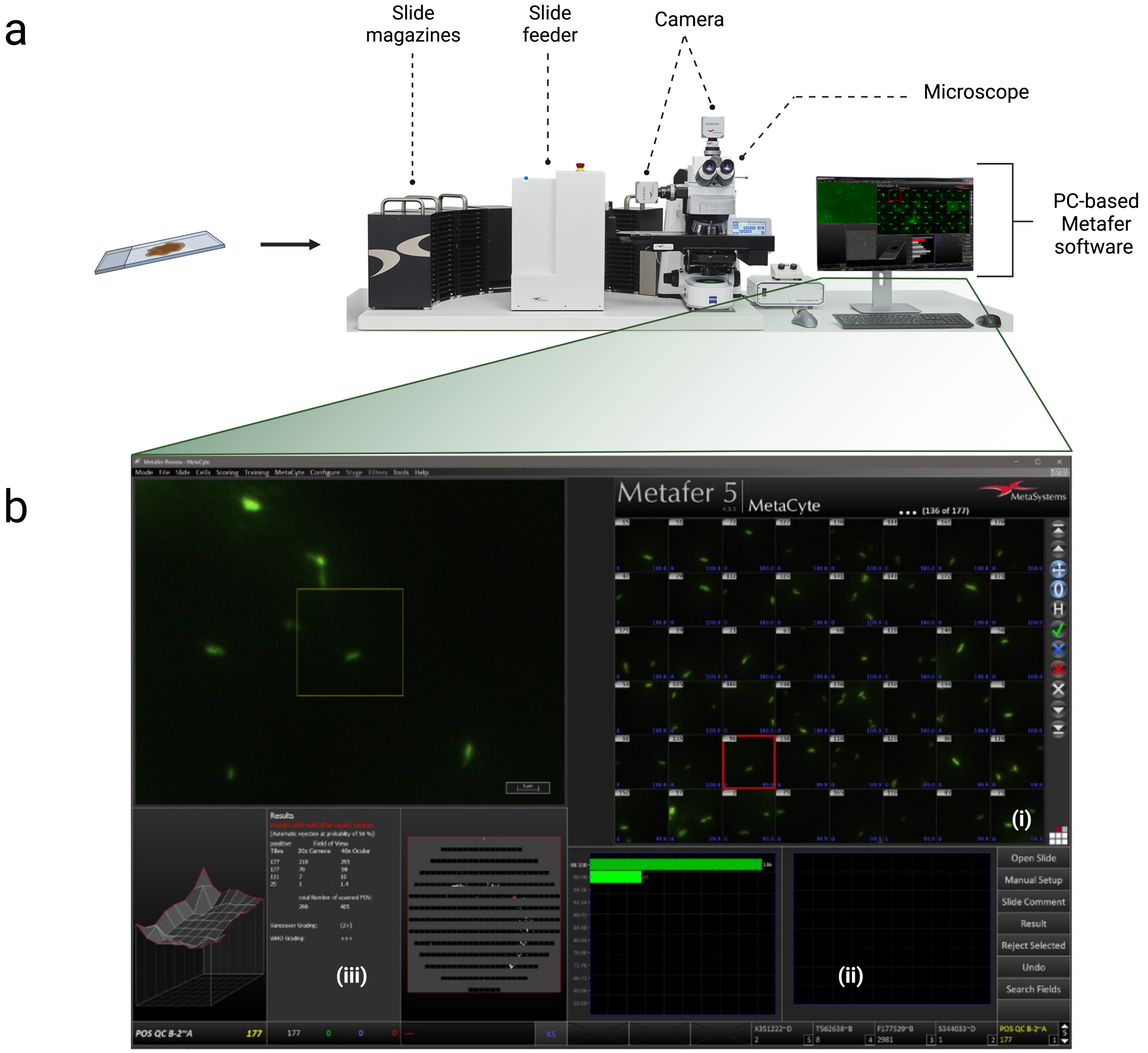

Figure S1. Automated digital microscopy workflow : (a) Components of the MetaSystems imaging and scanning platform include a fully automated microscope (Carl Zeiss Axio Imager Z2 fluorescent microscope equipped with 20X and 40X objectives) coupled with a high-resolution camera (CoolCube 4th Generation 12 MP camera 12Mega 1.1' CMOS Color Chip, pixel size 3.45 µm x 3.45 µm), a slide frame magazines and feeder, and a PC-based Metafer® software (1). **(b)** Analysis output generated by the Metafer Neon AFB module software (version 4.3.130) and displayed on a viewing monitor; the software segments a customizable number of scanned fields of view into small image tiles. Each tile is analyzed by a Deep Neural Network (DNN) algorithm pre-trained by the manufacturer to recognize objects suspicious of AFB. Tiles are sorted and displayed within an image gallery for review according to their probability of containing AFB-objects **(i);** based on a pre-determined DNN probability threshold (PT) (i.e., value that separate background/ artifacts from true AFB), the trained reviewer is also shown an overview of the distribution of probabilities assigned for all suspicious AFB-objects detected **(ii)**; an AFB smear status and grading score is proposed following tiles review **(iii)**.

Table S1. AFB grading reporting of auramine-O-stained slides for assisted-digital microscopy (a-DM) and manual microscopy (MM)

**CDC's proposed grading scale for reporting average number of AFB[a] seen using fluorescence microscopy (2)**		**MetaSystems grading scale based on average number of positive AFB-objects with PT ≥ 96%**	**Automated proposal**	
**At 200X magnification**	**At 400X magnification**		**Semi-quantitative AFB grading score**	**AFB smear status interpretation**
0 per slide	0 per slide[b]	0 per slide	No AFB seen	Smear-negative
1-2 per slide[c]	1-2 per slide[c]	1-2 per slide	Report exact count	Doubtful[d]
1-11 per 10 fields	3-18 per 50 fields	0.0034-0.017 per field	1+	Smear-positive
1-11 per field	4-36 per 10 fields	0.017-3.6 per field	2+	
10-113/ field	4-36 per field	3.6-36 per field	3+	
>113 per field	>36 per field	>36 per field	4+	

[a] Number of AFB observed per microscopic field.

[b] As per CDC, the minimum number of fields to examine before reporting an AFB-smear as negative is 55 at 400X magnification (2)

[c] As per CDC, counts of < 3 AFB are considered doubtful (3, 4).

[d] Slides with 1-2 AFB were reported as AFB smear-negative

Abbreviations: **AFB**, acid-fast bacilli; **no**.number; **PT**, probability threshold

Table S2. Summary of investigational steps in the event of discrepant AFB smear status results between both microscopic fluorescent modalities

First AFB smear examination		Alternate testing	Repeat examination AFB smear status result			Residual discrepancy
MM[a]	a-DM		MM after storage	ZN re-staining	a-DM	
Positive	Positive	No further testing				No
Negative	Negative	No further testing				No
Negative	Positive	▪ Repeat MM examination.<br>▪ Re-scan original slide with a-DM re-interpretation	Negative	NA	Negative	No
			Negative	NA	Positive	Yes
			Positive	NA	Negative	Yes
			Positive	NA	Positive	No
Positive	Negative	▪ Repeat MM examination<br>▪ Re-scan original slide with a-DM re-interpretation<br>▪ Re-stain original slides with ZN technique	Positive	Positive	Positive	No
			Positive	Negative[b]	Positive	No
			Positive	Positive	Negative	Yes
			Positive	Negative[b]	Negative	Yes
			Negative	Positive	Positive	No (Confirmed fading)
			Negative	Negative[b]	Positive	No [c]
			Negative	Positive	Negative	No (Confirmed fading)
			Negative	Negative[b]	Negative	No (Possible fading)

[a] Comparative method employed as standard fluorescent AFB smear method

[b] Sensitivity of fluorescence microscopy is higher than carbol fuchsin-stained microscopy and thus, it is possible that the presence of AFB on MM cannot be confirmed by overstaining using ZN, particularly for low AFB smear-positive slides (3, 5, 6).

[c] For slides originally reported as AFB-smear-positive by MM and with a grading score result of 1+, a repeat MM reading may yield a negative AFB-smear result due to either fading or presence of low AFB inoculum, and as a result, first MM examination was considered to assess presence residual discrepancy (7)

Abbreviations: **AFB**, acid-fast bacilli; **MM**: manual microscopy; **a-DM**: assisted-digital microscopy; **ZN**, Zielh-Neelsen; NA; non-available

Table S3a. Digital microscopy limit of detection for *Mycobacterium tuberculosis complex* (MTBC) and *Mycobacterium avium complex* (MAC)

**Dilution of organism stocks**[a]	**Approximate organism concentrations**[b]	**Number and percent of replicates positive for AFB**			
		**MTB H37Rv**		**MAC TMC 724**	
		**DM**	**MM**	**DM**	**MM**
1:16	12,313 CFU/mL	15/15 (100%)	5/5 (100%)	15/15 (100%)	5/5 (100%)
1:32	6,156 CFU/mL	15/15 (100%)	5/5 (100%)	14/15 (93%)	5/5 (100%)
1:64	3,078 CFU/mL	15/15 (100%)	5/5 (100%)	14/15 (93%)	5/5 (100%)
1:128	1,539 CFU/mL	9/30 (30%)	4/10 (40%)	14/30 (47%)	5/10 (50%)
1: 256	770 CFU/mL	0/15 (0%)	0/5 (0%)	0/15 (0%)	0/5 (0%)

[a] 1.0 McFarland standard

[b] Based on dilution of 1.0 McFarland standard with an approximate cell density of 3.0×10^6 CFU/mL (8, 9)

Table S3b. Repeatability and Reproducibility

**Sample description**	**AFB smear status and grading score**	**DM reproducibility**
Pleural fluid	Negative	3/3 (100%)
Pleural fluid	Negative	3/3 (100%)
Sputum	Negative	3/3 (100%)
Sputum	Negative	3/3 (100%)
Bronchial washing	Negative	3/3 (100%)
**Overall reproducibility AFB smear negative slides**		15/15 (100%)
Sputum	Positive (1+)	3/3 (100%)
Sputum	Positive (2+)	3/3 (100%)
Sputum	Positive (3+)	3/3 (100%)
Bronchial aspirate	Positive (4+)	2/3(66.7%)
Sputum	Positive (4+)	3/3 (100%)
**Overall reproducibility AFB smear positive slides**		14/15 (93%)

**AFB smear type**	**DM repeatability**			**DM reproducibility**
	**Day 1**	**Day 2**	**Day 3**	
AFB MTB positive[c]	5/5 (100%)	5/5 (100%)	5/5 (100%)	15/15 (100%)
AFB negative[d]	5/5 (100%)	5/5 (100%)	5/5 (100%)	15/15 (100%)

[c] 1: 64 dilution of 1.0 McFarland standard of MTB strain H37Rv

[d] Negative control (saline)

Table S4. Comparison of semi-quantitative AFB grading scoring between assisted digital microscopy and manual microscopy

Assisted-DM	Manual microscopy						Total
	No AFB seen (n= 201)	Doubtful (n=0)	1+ (n=28)	2+ (n=21)	3+ (n=14)	4+ (n=22)	
**No AFB seen**	181	0	8	1	0	0	**190**
**Doubtful**	9	0	3	2	0	0	**14**
**1+**	7	0	8	1	0	0	**16**
**2+**	4	0	9	17	9	9	**48**
**3+**	0	0	0	0	5	10	**15**
**4+**	0	0	0	0	0	3	**3**

Table S5. Investigations of discrepant results

Sample type	First AFB smear examination			Second AFB smear examination				Residual discrepancy	Mycobacterial culture	Final interpretation of a-DM result based on culture result
	MM	a-DM		MM		a-DM				
	**AFB smear status/ grading initially reported**	**Age of slides[a] (days)**	**AFB smear status/ grading initially reported**	**After storage**	**After ZN re -staining**	**Rescan and review at PT≥ 96%**	**Exploratory review[b] at PT≥ 80% and/or according to other manufacturer's parameters**			
BW	Negative	15	Positive (1+)	Negative	NA	Positive (2+)	NA	Yes	No growth	FP
BAL	Negative	9	Positive (2+)	Negative	NA	Positive (2+)	NA	Yes	No growth	FP
TA	Negative	9	Positive (1+)	Negative	NA	Positive (1+)	NA	Yes	No growth	FP
BAL	Negative	32	Positive (1+)	Negative	NA	Positive (1+)	NA	Yes	No growth	FP
BAL	Negative	15	Positive (1+)	Negative	NA	Positive (1+)	NA	Yes	No growth	FP
BAL	Negative	35	Positive (2+)	Negative	NA	**Doubtful**	NA	No	Growth of MAC	FN
PF	Negative	35	Positive (2+)	Negative	NA	Positive (2+)	NA	Yes	No growth	FP
Sputum	Negative	27	Positive (1+)	Negative	NA	Positive (2+)	NA	Yes	No growth	FP
Sputum	Negative	15	Positive (1+)	Negative	NA	Positive (1+)	NA	Yes	No growth	FP
Sputum	Negative	13	Positive (1+)	Negative	NA	Positive (1+)	NA	Yes	No growth	FP
BAL	Negative	30	Positive (1+)	**Doubtful**	Negative	Positive (1+)	NA	Yes	Growth of MTBC	TP
BAL	Positive (1+)	246	Negative	Positive (1+)	Negative	**Positive (1+)**	**Positive (1+)**	No	No growth~	FP
Sputum	Positive (2+)	276	Doubtful	**Negative**	Negative	Doubtful	Doubtful	Possible fading	Growth of *M. abscessus subspecies abscessus*	FN
BAL	Positive (2+)	276	Negative	**Negative ▲**	Negative ▲	NA	**Negative/ Positive (2+)**	Yes	Growth of *M. heckeshornense*	FN
Sputum	Positive (1+)	2	Negative	**Negative ▲**	Negative ▲	NA	**Doubtful/ Positive (1+)**	Yes	No growth	TN
BAL	Positive (1+)	110	Negative	**Negative**	**Positive**	Negative	Negative	Confirmed fading	No growth**	TN
Sputum	Positive (1+)	194	Negative	**Negative**	Negative	Negative	Negative	Possible fading	No growth~	TN
Sputum	Positive (1+)	189	Doubtful	**Positive (1+)**	Negative	**Positive (1+)**	**Positive (1+)**	No	Growth of MTBC	TP
Sputum	Positive (1+)	175	Negative	Negative	Negative	**Doubtful**	Negative	No	No growth	TN
Sputum	Positive (1+)	175	Negative	**Negative**	Negative	Negative	Negative	No	No growth∩	TN
Sputum	Positive (1+)	44	Negative	**Negative**	Negative	**Doubtful**	**Positive (1+)**	No	Growth of MAC	FN
Sputum	Positive (1+)	68	Negative	**Negative**	Negative	**Positive (1+)**	**Positive (1+)**	No	Growth of MTBC	TP
Sputum	Positive (1+)	237	Doubtful	**Negative**	Negative	**Positive (1+)**	**Doubtful**	No	No growth ᵀ	FP
Sputum	Positive (2+)	225	Doubtful	**Negative**	Negative	Doubtful	**Doubtful**	Possible fading	Growth of MTBC	FN
Sputum	Positive (1+)	21	Doubtful	**Negative ▲**	Negative ▲	NA	**Positive (2+)**	Yes	Growth of MTBC	FN

[a] Age of slide at initial scanning time
[b] Exploratory review of positive AFB-object seen during initial assisted-digital microscopy AFB examination at lower DNN probability threshold and/or according to parameters determined by the manufacturer.
In bold characters, results differing from previous examination same microscopic modality.
~ MTBC detected by PCR.

Highlighted slides sent to manufacturer to review. ▲ For this subset of slides, results from second MM AFB examination **followed** 2nd assisted DM AFB examination and both were not considered to resolve discordance.
** Patient known for MAC different specimen same day
∩ PCR negative for MTB and MAC

ᵀ Patient previously known for TB different specimen same day

Abbreviations: a-DM, assisted digital microscopy; **DM**, digital microscopy; **MM**, manual microscopy; **ZN**, Ziehl-Neelsen; **PT**, probability threshold; **TA**, tracheal aspirate; **NA**, non-applicable; **MAC**, *Mycobacterium avium* complex; **MTBC**, *Mycobacterium tuberculosis* complex; **TP**, true positive; **FP**, false positive; **FN**, false negative; **TN**, true negative

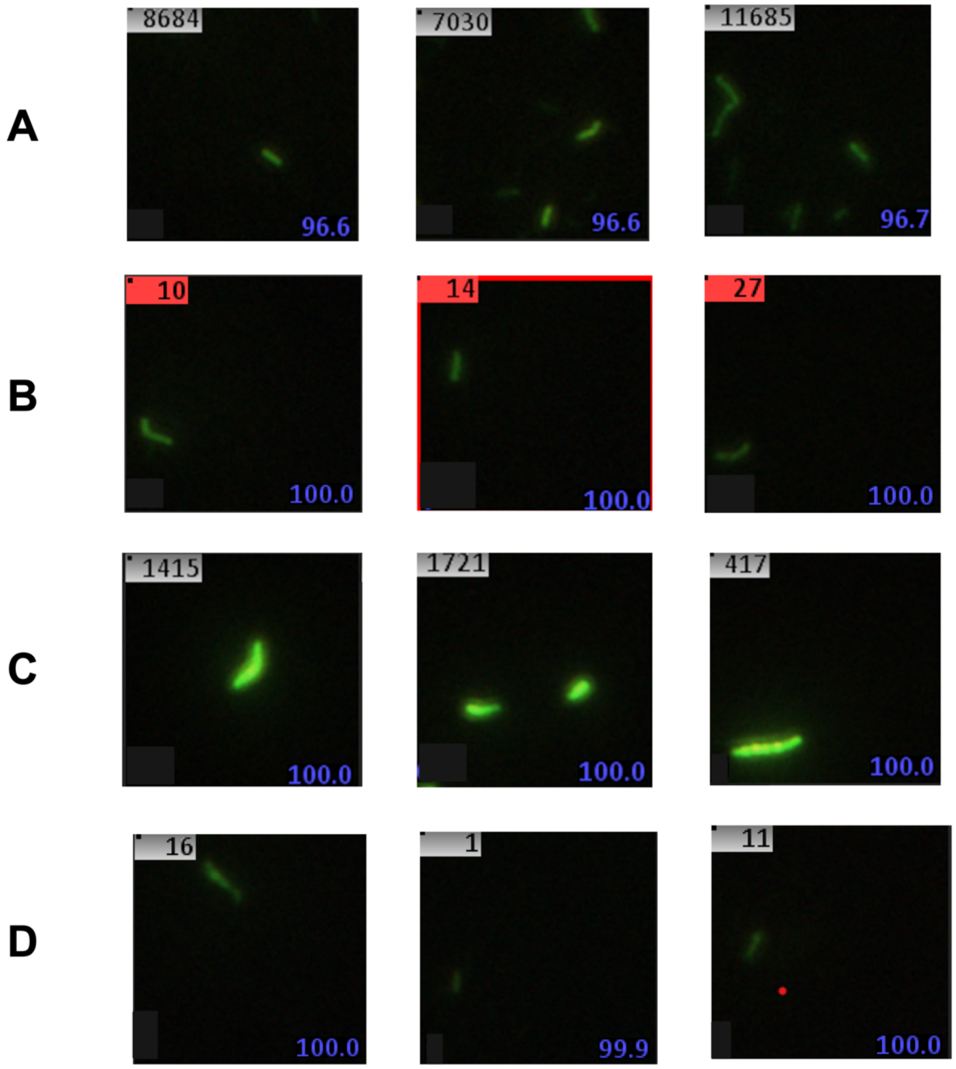

Supplementary Figure S2. Example of image tiles of AFB-objects detected at PT ≥ 96% displayed for review. **(a)** Concordant case: AFB-objects from a MM AFB smear positive slide associated with MTBC growth; **(b)** Discordant case: AFB-objects from a MM AFB smear negative slide associated with MTBC growth. **(c)** NTM concordant case: AFB-objects detected in a MM AFB smear positive with corresponding growth of MAC. **(d)** Major discordance case: AFB-object seen in a MM AFB smear negative with no growth in culture.

Upper left number: Tiles number per the software

Blue lower right number: The probability of representing a true AFB in the respective tile as per software DNN.

Abbreviations: **AFB**, acid-fast bacilli; **MTBC,** *Mycobacterium tuberculosis* complex, **MM**, manual microscopy; MAC, *Mycobacterium avium* complex; **a-DM**, assisted digital microscopy.

REFERENCES SUPPLEMENTAL MATERIAL

1. Horvath L, Hanselmann S, Mannsperger H, Degenhardt S, Last K, Zimmermann S, Burckhardt I. 2020. Machine-assisted interpretation of auramine stains substantially increases through-put and sensitivity of microscopic tuberculosis diagnosis. Tuberculosis (Edinb) 125:101993.
2. CLSI. 2018. *Laboratory Detection and Identification of Mycobacteria; Approved Guideline.* CLSI document M48-A2 Wayne, PA : Clinical and Laboratory Standards Institute; 2018.
3. Forbes BA, Hall GS, Miller MB, Novak SM, Rowlinson MC, Salfinger M, Somoskovi A, Warshauer DM, Wilson ML. 2018. Practical Guidance for Clinical Microbiology Laboratories: Mycobacteria. Clin Microbiol Rev 31.
4. Kent P, Kubica GP. 1985. Public health mycobacteriology: a guide for the level II laboratory. US Department of Helath and Human Services Centers for Disease Contriol.
5. Steingart KR, Ramsay A, Pai M. 2007. Optimizing sputum smear microscopy for the diagnosis of pulmonary tuberculosis. Expert Rev Anti Infect Ther 5:327-31.
6. Laboratories AoPH. 2019. Mycobacterium tuberculosis : Assessing Your Laboratory. https://www.aphl.org/aboutAPHL/publications/Documents/ID-2019Apr-TB-Toolkit.pdf. Accessed 25 July 2023.
7. Aziz MA, Association of Public Health Laboratories (U.S.);Centers for Disease Control and Prevention (U.S.);International Union against Tuberculosis and Lung Disease;World Health Organization. 2002. External quality assessment for AFB smear microscopy. https://stacks.cdc.gov/view/cdc/11440. Accessed July 25, 2023.
8. Penuelas-Urquides K, Villarreal-Trevino L, Silva-Ramirez B, Rivadeneyra-Espinoza L, Said-Fernandez S, de Leon MB. 2013. Measuring of Mycobacterium tuberculosis growth. A correlation of the optical measurements with colony forming units. Braz J Microbiol 44:287-9.
9. CLSI. 2018. Susceptibility Testing of Mycobacteria, Nocardia spp., and Other Aerobic Actinomycetes. 3rd ed. . CLSI standard M24 Wayne, PA: Clinical and Laboratory Standards Institute; 2018.
